# Rethinking Basic Science Teaching in Medical Education: A Viewpoint on Its Past, Present, and Future

**DOI:** 10.2196/80646

**Published:** 2026-07-28

**Authors:** Kátia M Avena, Ana Paula A Brito, Luiz F Quintanilha, Mariana Araújo-Pereira, Bruno B Andrade

**Affiliations:** 1 Instituto Monster de Ensino, Assistência, Pesquisa e Desenvolvimento Tecnológico em Saúde, Av. Luís Viana Filho, 3230Imbuí, Salvador, Bahia, 41720-200, Brazil, +55 71 3511-4840; 2 Clinical and Translational Research Laboratory / Instituto Gonçalo Moniz, Fundação Oswaldo Cruz, Salvador, Bahia, Brazil; 3 Academic Operations, Clariens Educação, Salvador, Bahia, Brazil; 4 Universidade do Estado da Bahia, Salvador, Bahia, Brazil; 5 School of Medicine, Faculdade Zarns Salvador, Salvador, Bahia, Brazil; 6 Graduate Program in Regional and Urban Development, Salvador University, Salvador, Brazil; 7 Division of Infectious Diseases, Johns Hopkins Medicine, Baltimore, MD, United States; 8 Department of International Health, Bloomberg School of Public Health, Johns Hopkins Medicine, Baltimore, MD, United States

**Keywords:** medical education, medical schools, medical training, basic sciences, technology, pedagogy

## Abstract

Basic sciences remain a cornerstone of medical education, but the terms of the debate about their importance have changed. The central risk is no longer their formal exclusion from medical curricula but their curricular invisibility: the gradual dilution of mechanistic reasoning within integrated, technology-mediated, and institutionally unequal training environments. In this Viewpoint, we revisit the past, present, and future of basic science teaching to argue that reform must protect conceptual progression, curricular visibility, and clinical transfer. Active learning, curricular integration, digital tools, and AI may strengthen scientific literacy, but only when they are anchored in faculty development, institutional support, and explicit commitments to rigor and equity.

## Introduction

The teaching of basic sciences has long occupied a central place in medical education, providing the theoretical basis for clinical reasoning and professional practice [1]. Disciplines such as anatomy, biochemistry, and physiology have traditionally supported medical training by helping students understand the structure and function of the human body in health and disease [2,3]. In this article, the term “basic sciences” refers to biomedical disciplines that provide the conceptual and mechanistic basis for understanding normal and pathological processes and for interpreting clinical phenomena. Given their historical role as a core component of medical curricula, revisiting how these disciplines are taught has become increasingly necessary, particularly in light of contemporary educational and health care challenges [2-4]. This discussion is especially important in the current context of curricular reform, in which efforts to improve relevance, integration, and efficiency may also, sometimes unintentionally, weaken the visibility, coherence, or depth of foundational scientific knowledge.

This challenge is particularly evident across many settings in the Global South, where the rapid expansion of medical education has often occurred alongside resource constraints, uneven institutional capacity, and, in some contexts, increasing privatization, raising concerns about training quality and professional preparedness [5-7]. In such settings, the central issue is no longer whether basic sciences remain important but how to preserve their role within increasingly complex, technology-mediated, and unequal educational settings. At the same time, the continuous evolution of medical knowledge and the growing complexity of health care have required substantial changes in how these disciplines are taught [3]. The task is not simply to modernize teaching but to do so without weakening the scientific foundations of medical training. As curricula move toward integration, competency-based education, and digital innovation, basic sciences may either be meaningfully recontextualized or progressively marginalized. This marginalization does not necessarily mean that basic sciences are formally removed from the curriculum. More often, it occurs when they remain present but become fragmented, are superficially addressed, and are less visible as the conceptual basis for clinical reasoning within integrated curricula. This tension lies at the center of contemporary medical education: how to make the teaching of basic sciences more relevant, practical, and student-centered while preserving the rigor, depth, and conceptual coherence that sustain clinical reasoning and professional judgment.

This Viewpoint is a critical reflection grounded in institutional experience and supported by selected literature. Its purpose is not to review every model of basic science teaching but to clarify the conditions under which reform becomes educationally meaningful. Integration, active learning, and digital innovation matter only when they make the scientific basis of clinical reasoning more visible, more usable, and more durable throughout the curriculum.

The contribution of this article is therefore conceptual. We argue that the main threat to basic science education is not its absence from medical curricula but rather its curricular invisibility. Contemporary debates do not usually call for the formal removal of basic sciences from medical curricula. More often, they question the traditional basic-clinical distinction [3] or advocate the stronger dissolution of the preclinical phase into clinically oriented teaching [8]. The concern lies neither in integration itself nor in efforts to align basic sciences with clinical practice. On the contrary, such initiatives can strengthen medical education when they are well designed. The problem arises when reform weakens the conceptual progression of basic sciences, their curricular visibility, and their interpretive role within integrated curricula. This risk becomes more pronounced when technology is adopted more rapidly than pedagogy, when faculty development is treated as optional, and when educational expansion occurs without commensurate investment in infrastructure and quality assurance. The past-present-future structure is used here to show how this problem emerged, how it currently appears, and what must be preserved if reform is to strengthen rather than weaken the foundational and scientific training of physicians.

## The Past: The Historical Legacy of Basic Science Teaching in Medical Education

The modern place of basic sciences was consolidated during the broader transformation that made medical education more scientific, structured, and institutionally grounded [9-11]. The Flexner report [11] and the Dawson report [12] matter here less as historical landmarks than as sources of a tension that is still present: medical education gained scientific legitimacy, but the curricular form used to protect that legitimacy often separated mechanism from clinical use.

The Flexner report [11] strengthened the expectation that clinical practice should rest on foundational scientific knowledge [2,13,14]. Yet the curriculum that followed was commonly lecture-based, discipline-centered, and dependent on memorization and laboratory instruction. The result was productive and limiting at the same time. It gave basic sciences authority, but it also helped place them in a preclinical space that students could experience as distant from patient care. The Dawson report [12], by emphasizing the relation between education, service, and clinical context, pointed to a different possibility, but the compartmentalized model remained dominant.

Throughout much of the 20th century, basic science teaching remained organized around passive transmission, delayed application, and limited clinical contextualization [15,16]. This approach helped consolidate foundational knowledge, but it also encouraged a form of learning in which detail could accumulate without becoming reasoning. The problem became visible across anatomy, biochemistry, and microbiology, where students often encountered scientific content before they understood why it mattered for diagnosis, prognosis, or therapeutic judgment [17-22].

The historical lesson is therefore precise. The basic sciences were protected by a model that also isolated them. Current reforms still carry this inheritance: they must reduce fragmentation and excessive content load without dissolving the conceptual progression that allows foundational knowledge to become clinical reasoning [23]. The task is not to move away from science but to move scientific explanation closer to the work of interpretation, judgment, and care.

## The Present: Current Practices and Challenges in Basic Science Education

Basic science teaching in medical education is currently marked by both important advances and persistent tensions. In many schools, disciplines such as anatomy, physiology, biochemistry, pharmacology, and microbiology are being restructured to strengthen the relationship between foundational knowledge and clinical application. This movement reflects broader efforts to move away from fragmented, content-heavy models and toward curricula that are more integrated, interactive, and responsive to contemporary health care needs. However, this transformation remains incomplete, especially in settings where infrastructure is limited, institutional support is weak, or resistance to pedagogical change remains strong.

A central feature of this transition has been the attempt to overcome the longstanding divide between theory and practice. Traditional models, heavily centered on content accumulation and memorization, often produced cognitive overload and limited students’ ability to connect foundational concepts with clinical reasoning [8,24]. In response, many medical schools have adopted more integrated approaches, including problem-based learning (PBL), case-based teaching, and spiral curricula, all of which seek to connect basic and clinical sciences more meaningfully [13,25,26]. Recent work in physiology education has also reinforced the value of active learning strategies in basic science teaching [27]. At the same time, curricular reforms have increasingly emphasized the horizontal and vertical integration of the basic sciences across the curriculum, supported by active learning strategies and faculty development efforts aimed at strengthening integration between basic and clinical sciences [28].

Clinically oriented integration may also support retention. Earlier clinical exposure, clerkship-based reinforcement, and integrated assessment have been associated with stronger preservation of foundational knowledge [28,29]. In a recent single-institution study from Sudan, students performed better on integrated questions than on discipline-specific items, suggesting that basic science knowledge is more durable when students must use it to interpret clinical problems [29]. This evidence supports competency-based education, provided that competence is understood as reasoning with science rather than merely performing clinical tasks [30,31].

Even so, progress remains uneven. Many institutions continue to struggle with insufficient pedagogical support and limited investment in faculty development [32,33]. Educators trained in more traditional systems may find it difficult to adopt active methods, integrate theory with clinical reasoning, or use digital tools effectively [34-36]. Evidence also suggests that even when faculty value active learning more highly, traditional lectures often remain part of their routine, highlighting a possible mismatch between pedagogical beliefs and teaching practices [36]. This gap between curricular intention and classroom reality shows that reform depends not only on curricular redesign but also on preparing teachers to interpret and enact new models consistently. These barriers are especially relevant in contexts where nonphysician educators are responsible for much of the teaching in the basic sciences. Although these faculty members often bring strong disciplinary knowledge and considerable pedagogical potential, they may receive limited institutional recognition or support, which can hinder innovation and interdisciplinarity [37].

These tensions reveal a broader challenge in contemporary medical education: how to modernize teaching without weakening scientific rigor. Making basic science education more relevant does not mean reducing its depth but rather teaching it in ways that are more contextualized, clinically meaningful, and educationally effective. Real clinical cases, cross-disciplinary dialogue, and stronger collaboration between basic and clinical faculty can help students understand how foundational knowledge informs medical judgment and patient care. In this sense, the goal is not to replace the basic sciences but to reposition them more effectively within the curriculum.

Interdisciplinarity has become one of the clearest expressions of this repositioning. Contemporary curricula increasingly incorporate social sciences, public health, and ethics into the teaching of basic disciplines, reflecting the complexity and interconnectedness of modern health care [8,38]. This broader framework supports more patient-centered and socially responsive training while also helping students connect scientific knowledge with the human, ethical, and systemic dimensions of practice. Emerging approaches such as STEAM (science, technology, engineering, arts, and mathematics) may further enrich this process by encouraging interdisciplinary projects, creativity, empathy, and visual reasoning [39]. The approach’s emphasis on collaboration, design, and real-world problem-solving can help humanize scientific training while preserving rigor. In medical education, this approach is particularly valuable because it reinforces the idea that science and human experience should not be taught as separate domains.

Technological innovation has also reshaped current teaching practices [40-42]. After the COVID-19 pandemic, digital tools became even more prominent in health professions education [43,44]. In anatomy, cadaveric dissection is now frequently complemented by 3D models, virtual dissections, imaging-based simulations, and, in some contexts, virtual reality [17,18]. These tools can expand access, reduce dependence on costly physical resources, and offer flexible opportunities for visualization and repetition. Although cadaveric dissection retains important symbolic and educational value, newer strategies have broadened the possibilities for anatomy teaching. Of note, technology is most effective when it supports conceptual understanding, guided interpretation, and clinical transfer rather than functioning merely as a substitute for traditional materials or as an isolated innovation.

Similar changes are evident in other disciplines. In histology, pathology, and microbiology, virtual laboratories and digital microscopy have shown educational value while also reducing ethical and logistical concerns related to animals and biological materials [45-49]. In biochemistry, a field often perceived as abstract, gamification, simulation, and clinically oriented cases have made learning more engaging and relevant [50,51]. These innovations make learning more meaningful. Given the direct clinical implications of biochemical knowledge, active and contextualized approaches are particularly important in this area [52,53]. Physiology has also evolved through the use of digital simulations and gamified environments that connect normal and abnormal function while reducing reliance on animal-based teaching in nonclinical settings [54,55]. Across these examples, the underlying issue is not simply the adoption of new tools but the effort to make foundational scientific knowledge more accessible, meaningful, and applicable.

Yet these innovations do not affect all institutions equally. The expansion of online and technology-enhanced education has increased flexibility, autonomy, and opportunities for personalized learning, but it has also deepened inequalities between institutions and regions [7,56]. Many schools, especially in low- and middle-income countries (LMICs), still lack reliable internet access, adequate equipment, or sufficient technical and pedagogical support. This digital divide threatens equity and inclusion and reinforces the idea that innovation without investment may widen rather than reduce disparities [4].

Faculty development is therefore not an accessory to reform; it is the condition that makes reform possible [57]. Teachers need support to design active learning, construct assessments that reward reasoning, connect basic mechanisms to clinical decisions, and use digital tools without allowing the tool to become the lesson [37]. AI literacy now belongs in this preparation, but it should be framed critically: educators must learn where AI can reduce routine work and where human judgment, feedback, and mentorship remain irreplaceable. This support must include physician and nonphysician educators because both carry the responsibility for making science visible within medical training.

Structural problems also remain highly relevant. In many settings in the Global South, medical schools continue to face curricular fragmentation, inadequate infrastructure, faculty shortages, and uneven regulatory capacity, all of which compromise training quality [5,6,58]. In some contexts, these challenges have been further intensified by the rapid expansion of medical education without proportional investment in educational resources, faculty support, or quality assurance mechanisms. Increasing privatization, weak regulation, and poor coordination between education and health care systems have further widened disparities and left many curricula insufficiently aligned with real health care needs [6,7,58-61].

The present moment is therefore defined by a difficult coexistence. Innovation is real, but so is inertia. Integration, interdisciplinarity, and technology have improved parts of basic science education, while structural weakness still determines how far these gains can extend. Preserving the basic sciences now requires more than new tools or new language. It requires institutional choices that protect scientific depth, make foundational knowledge clinically actionable, and reduce the gap between well-resourced and underresourced schools.

## The Future: Emerging Trends and Future Perspectives in Basic Science Education

The future of basic science education will be shaped less by the availability of new technologies than by the judgment used to place them within the curriculum. AI, augmented reality, virtual reality, and high-fidelity simulation can make learning more adaptive and immersive. They can also create another layer of distraction if they are adopted as symbols of modernization rather than as instruments of understanding. Future innovation must therefore be measured by a simple standard: whether it helps students reason more clearly from mechanism to clinical consequence. Concerns about digital fatigue and screen-mediated disconnection make this standard even more important, since medical education still depends on attention, dialogue, mentorship, and relational learning [62,63].

AI offers important possibilities for personalized education, adaptive learning, and scalable instructional support in medical training [64]. Adaptive systems can analyze student performance in real time, provide targeted feedback, and help address knowledge gaps more quickly. These functions may improve learning efficiency. However, the role of AI in replacing teaching competencies remains uncertain. A recent study with educators and technology experts found agreement only in specific areas, particularly those involving low-complexity tasks such as preparing materials or grading [65]. In contrast, linking theory to practice, anticipating student needs, and adjusting strategies were seen as activities that still depend on human judgment. Empathy and critical insight were also identified as irreplaceable aspects of teaching.

These findings suggest that AI can support repetitive tasks but not replace human guidance. Medical education continues to require emotional awareness, empathy, and active listening [66]. At a time when concerns about screen fatigue and digitally mediated disconnection are becoming more visible, preserving human interaction in educational settings becomes even more important [62,63]. In this context, the educator’s role is shifting from content delivery to learning facilitation. Teachers are increasingly expected to act as mentors who guide critical thinking and ethical reflection while helping students use technology wisely and maintain human values at the center of education [66]. The educator of the future will therefore need to combine disciplinary expertise, pedagogical judgment, digital literacy, and the ability to sustain reflective and relational forms of learning in increasingly technology-rich environments.

Virtual and augmented reality are also reshaping learning. These technologies create highly interactive environments in which students can explore anatomy and physiology through 3D simulations. Such experiences can improve spatial reasoning and decision-making in clinical scenarios while allowing learners to practice safely before engaging with real patients. However, their expansion should complement, rather than displace, the relational dimensions of medical education, particularly in areas where communication and professional identity depend on interaction with teachers, peers, and patients [67].

Future curricula will likely continue to emphasize collaboration, interdisciplinarity, and competency-based learning. This broader scope can bring ethics, social sciences, and digital health closer to basic sciences, but only if integration preserves the logic of scientific explanation. Memorization should give way to application, reflection, and adaptability, yet application without a foundation becomes fragile. The value of basic sciences lies in their capacity to support reasoning, interpretation, and decision-making across clinical and social contexts rather than in the isolated accumulation of facts.

These changes point to a broader structural transformation. Basic science education is moving from rigid and fragmented models toward curricula that integrate disciplines, technology, and human development. Table 1 summarizes these shifts across pedagogical approaches, curricular structure, teaching tools, engagement, assessment, and clinical integration. The table should be read as a map of pressures rather than a prediction of automatic progress. Equity will decide whether these innovations become shared educational gains or another mechanism of stratification. In low- and middle-income settings, aspirations toward innovation often coexist with unstable connectivity, limited simulation resources, constrained faculty capacity, and uneven regulatory support.

**Table 1.** Summary comparison of past, present, and future trends in basic science teaching in medical education.

Aspects	Past trends	Present trends	Future trends
Pedagogical approach	Teacher-centered lectures and passive learning	Student-centered active learning, including problem-based learning and team-based learning	Personalized and adaptive learning used to strengthen reasoning and feedback
Curricular structure	Isolated disciplines with minimal integration across areas	Integrated, system-based, and interdisciplinary curricula	Competency-based curricula with explicit progression of basic science concepts
Teaching tools	Textbooks, cadaveric dissection, and chalkboards	Cadaveric dissections with virtual reality, augmented reality, online platforms, and simulations	Immersive simulation; AI-supported tutoring; and science, technology, engineering, arts, and mathematics–informed resources tied to clear learning goals
Student engagement	Passive learning focused on memorization	Active participation, problem-solving, and collaborative learning	Autonomous learning paths supported by guided feedback and clinically grounded problem-solving
Assessment methods	High-stakes examinations and rote memorization	Formative assessments, objective structured clinical examinations, and competency-based evaluations	Continuous assessment of reasoning, knowledge transfer, and reflective clinical application
Integration with clinical practice	Limited and delayed clinical application	Early clinical exposure and integration of clinical cases	Longitudinal integration of basic and clinical sciences while preserving conceptual visibility

Faculty development will remain critical in this future landscape [68]. Advanced technologies will not improve learning by themselves. Educators must know how to translate complex disciplinary content into clinical meaning, how to design feedback around reasoning, and how to decide when a digital tool adds value and when it distracts from the concept being taught. In basic science education, sustainable reform depends on this pedagogical judgment.

Students will also need to adapt. Active learning and technology-based education require new attitudes toward learning. Students must take greater responsibility for their own progress and develop autonomy, critical thinking, and resilience [69,70]. Success in this context depends on their ability to seek knowledge independently and apply it in dynamic situations. Consequently, the focus shifts from passive absorption to active exploration, supported by strategies that encourage self-regulation and lifelong learning. On the other hand, expectations of autonomy should be balanced with adequate guidance, feedback, and structured support, since students differ considerably in their readiness for self-directed learning and in their ability to navigate complex educational environments.

At the same time, the future of basic science education is threatened by erosion that can be difficult to see. Course hours may shrink, practical experiences may disappear, research requirements may be removed, and student-faculty ratios may grow while the curriculum still appears formally integrated [71]. The consequence is a thinner scientific literacy. Innovation will produce lasting benefit only if it protects the time, faculty, and pedagogical structure required for students to understand mechanisms deeply enough to use them.

A sustainable future for basic science education depends on political will, equitable investment, and cultural change within institutions. Technology can help, but it cannot compensate for weak pedagogy, undervalued faculty, or inadequate infrastructure. The central challenge is to modernize teaching while protecting the scientific and human foundations of physician training across diverse educational realities.

## Conclusions

Basic science education is being transformed by integration, active learning, and technology. These changes can improve medical training, but they can also make foundational knowledge less visible if reform is guided by novelty rather than by educational purpose. Across the past, present, and future of medical education, the central question is no longer whether basic sciences matter. It is whether medical schools can preserve the conceptual progression through which anatomy, physiology, biochemistry, microbiology, pharmacology, and pathology become clinical reasoning.

Defending the basic sciences does not mean returning to isolated preclinical courses or resisting curricular integration. It means refusing a weaker bargain in which science remains present in name but loses depth, sequence, and interpretive power. Integration is valuable when it makes mechanisms clinically intelligible. Technology is valuable when it deepens understanding rather than merely replaces attention. Faculty development is valuable when it equips teachers to connect explanation with judgment. The future of medical education will depend on this alignment: innovation that is strong enough to modernize training and scientific literacy that is strong enough to sustain the physicians who will practice in conditions of uncertainty.

## Abbreviations

LMIC: low- and middle-income country
PBL: problem-based learning
STEAM: science, technology, engineering, arts, and mathematics
